# An interview with Mingjie Zhang: phase separation in biological systems

**DOI:** 10.1093/nsr/nwab081

**Published:** 2021-05-01

**Authors:** Weijie Zhao, Mu-ming Poo

**Affiliations:** NSR news editor based, Beijing; Director of the CAS Center for Excellence in Brain Science and Intelligence Technology, and Executive Editor-in-Chief of NSR

## Abstract

Mingjie Zhang is a distinguished structural biologist whose research interest is the protein assemblies in cells, particularly those at the synapse, where two neurons meet and transmit nervous impulses from one to the other. In recent years, his group discovered the phenomenon of phase separation in both the pre- and post-synaptic sites of neurons and elucidated its regulation and physiological function. In this interview, *NSR* Executive Editor-in-Chief Mu-ming Poo talks with Prof. Zhang on the fast-developing research area of biological phase separation, as well as Zhang's new role as the Founding Dean of the School of Life Sciences at Southern University of Science and Technology (SUSTech) in Shenzhen.

## BIOLOGICAL PHASE SEPARATION

### Concept

***Poo:*** Phase separation has become one of the hottest topics in cell biology. Why?

***Zhang:*** Phase separation is a well-established phenomenon in physical sciences, and has been observed for a long time in biology. More than 100 years ago, Ramón Cajal discovered condensed bodies in the nucleus, which are now known as Cajal bodies and their formation is a phase separation process.

The 2009 paper published by Anthony Hyman and Clifford Brangwynne [Brangwynne C *et al. Science* 2009; 324: 1729–32] is probably the landmark paper that made biological phase separation a hot topic. They showed that in the developing *C. elegans* embryo, P granules form in a certain region via phase separation and this process is important for embryo development, making a connection between phase separation and physiological functions. Then suddenly, many people began to think that more biological phenomena that have been observed may be linked to phase separation.

***Poo:*** What exactly is biological phase separation?

***Zhang:*** In cells or other biological systems, biological molecules, such as proteins and nucleic acids, are able to spontaneously assemble into a state with lower entropy or higher order, forming a thermodynamic equilibrium with a condensed phase and a dilute phase co-existing in a system. That is the phenomenon of biological phase separation.

The phenomenon is exactly the same as when you pour black vinegar into olive oil. You will see beautifully separated two liquid phases. But in biological systems, the beauty is that the process happens in aqueous solution under biological conditions—neutral pH, as well as physiological salt and biomolecule concentrations.

Importantly, some of the condensed phases have biological functions and are considered to be non-membranous

**Figure 1:**
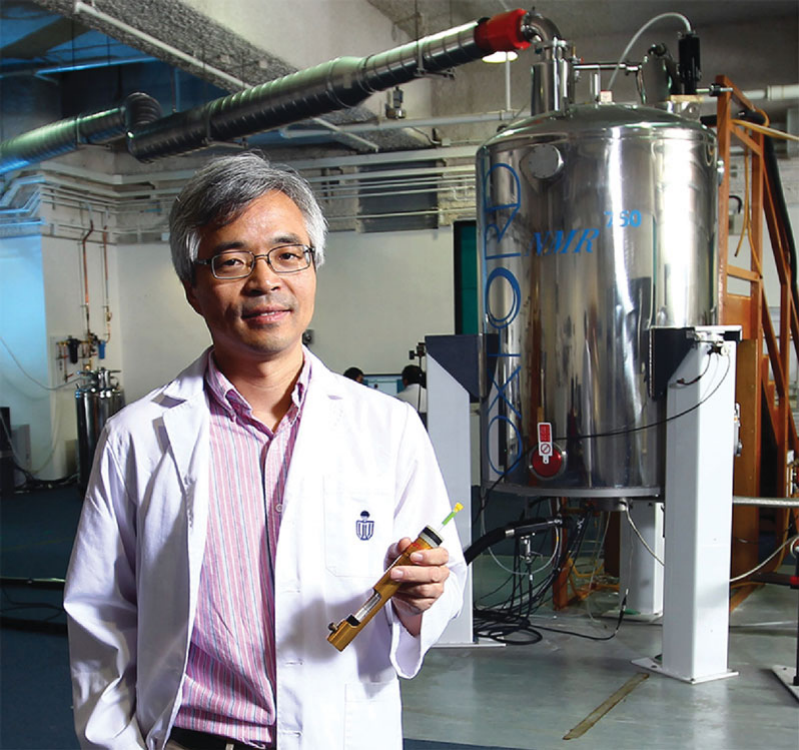
Structural biologist Mingjie Zhang is the Founding Dean of the School of Life Sciences, SUSTech *(Courtesy of Prof. Zhang)*.

organelles. And these organelles can interact or communicate with the membranous organelles, thus greatly expanding the number of organelles that a cell can use.

***Poo:*** In biology, we have learnt about lipid phase separation within the cell membrane for a long time. We knew that cholesterol can be enriched in one domain to form a ‘lipid raft’. But phase separation occurring in aqueous cytoplasm is a relatively new idea.

***Zhang:*** That's right. In the past, it has been a little bit difficult to imagine that soluble cytoplasmic molecules can condense into droplet-like phases.

***Poo:*** Toxic protein aggregates are relevant to various diseases. Do we consider the formation of those aggregates to be phase separation?

***Zhang:*** In the broad sense, you can consider it as phase separation. I think there are two general categories of phase separation—physiological phase separation that is functional and highly reversible, and the non-reversible phase separation that will very likely lead to functional defects. Disease-related aggregates of beta amyloids or short peptide repeats-containing proteins (e.g. polyQ, polyGR, polyPR, etc.) belong to the category of non-reversible phase separation, sometimes referred to as phase transition.

***Poo:*** What is the major driving force of biological phase separation, specific inter-molecular interaction or non-specific interaction?

***Zhang:*** There are actually two views on this issue. Some people have been saying that phase separation is driven by intrinsically disordered proteins. Another camp, which is relatively a minority now but is also my view, is that biological phase separation is driven by the combination of specific molecular interactions as well as non-specific interactions among intrinsically-disordered-sequence-containing proteins. From a thermodynamic point of view, intrinsically disordered proteins provide the general interaction driven by free energy gain, while specific molecular interactions provide specificity of condensates formation.

According to polymer chemistry theory, any polymer, including proteins, nucleic acids and their mixtures, is able to undergo phase separation under certain conditions. But in the biological systems, specific interactions are needed to drive the formation of condensed phases under physiological conditions. The specific interactions often involve multiple interaction sites and can form a network of molecules. When the network reaches a certain complexity, phase separation will happen spontaneously. Also, specific interactions can provide more space for the regulation of the condensed phases.

***Poo:*** Biological systems need energy input to maintain their structural order and functions. Do the formation and regulation of phase separation need energy input?

***Zhang:*** Phase separation *per se* does not need energy input. It happens spontaneously once the biomolecule concentration reaches a certain threshold. But the synthesis and turnover of the biomolecules, which build up the concentration, need energy. It's the same for regulation. The modification or concentration change of certain biomolecules needs energy input, and after that, the modulation of the condensed phase formation or dispersion would happen spontaneously.

***Poo:*** Phase separation is useful for cells because it can enrich certain molecules to a specific location. But if this localization

Phase separation is a very good way for cells to increase the efficiency and specificity of signal transduction. It will be surprising if cells do not use this kind of strategy more often than we have realized.

—Mingjie Zhang

**Figure 2:**
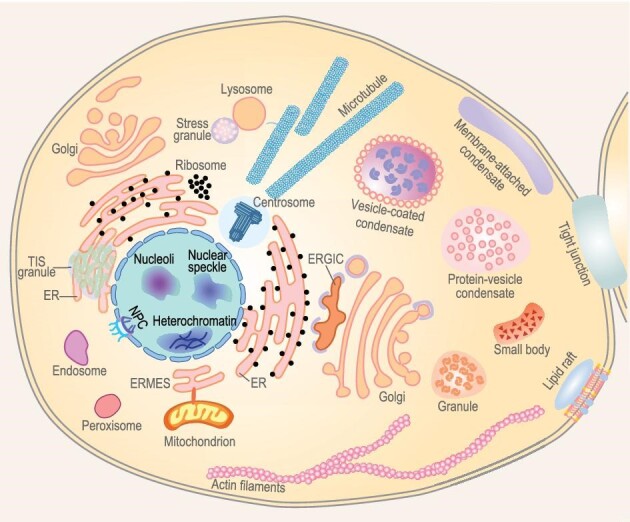
Phase separation in cell, showing the interactions and communications among membranous organelles and non-membranous organelles. Depicted by Xiandeng Wu.

process can be accomplished merely by specific molecular interactions, then why do cells need phase separation?

***Zhang:*** That's a really great question. The point is that, if the molecules are purely localized and enriched by specific interactions, their distribution would be governed by the diffusion law, meaning that there will be a concentration gradient throughout the cell. That means the cell has to synthesize a large amount of molecules in order to fulfill the requirement in some specific locations. But with phase separation, the concentration difference between the two phases can be as large as several hundred-fold, or even larger, so we do not need that many molecules to be synthesized. This is just one example. There are many features of biological condensates formed via phase separation and these features cannot be explained by the canonical stoichiometric and specific molecular interactions occurring in dilute solution.

### Examples

***Poo:*** Would you give some examples of biological phase separation?

***Zhang:*** One famous example is that in a fertilized egg, maternal and paternal materials are deposited in very condensed phases and are located in specific areas within the egg. That is important for embryo development.

Another example is, in a 2002 paper [Galkin O *et al. PNAS* 2002; 99: 8479–83], the authors talked about hemoglobin phase separation in red cells. They found that with gene mutation in sickle cell anemia patients, hemoglobin molecules undergo liquid-liquid phase separation much more easily and are more likely to form polymers that are found in sickle cells. That was a beautiful paper that linked phase separation with human diseases.

In the nucleus, one theory is that the partitioning of chromatin, or the formation of euchromatin and heterochromatin, is a process of phase separation. But there is strong resistance to this theory and we are still unsure if it's a real phase separation process or not. I think if it's proven to be true, it will provide many new angles in understanding chromosome organization and regulation.

***Poo:*** You had been a structural biologist and bumped into phase separation at the synapse in recent years. How did this happen?

***Zhang:*** In the last 25 years I have been trying to understand how synapses form and work. When looking at the electron microscope pictures of the synapse, you can see a high density of protein assembly right beneath the postsynaptic membrane. This protein assembly, which is called postsynaptic density (PSD), is so stable that it can be chemically purified, and so dynamic that it can be modulated by changes in synaptic activity. I really wanted to understand the chemical, structural and physical basis of the formation and regulation of PSD, and that has been the central theme of research in my lab.

We meticulously studied the molecular interactions of all major PSD proteins. When we were studying the interaction between PSD-95 and SynGAP, two major proteins in PSD, one of my students told me that when mixing these two proteins, some droplet-like phenomenon was observed. That was really a surprise and we suddenly realized that it may be a phase separation process that drives the formation of PSD. That was our initial discovery [Zeng M *et al. Cell* 2016; 166: 1163–75].

***Poo:*** Would you explain more about PSD?

***Zhang:*** PSDs were observed as electron-dense thickenings beneath postsynaptic plasma membranes and thus are open to the cytoplasm of dendritic spines. PSD contains many different proteins, and these proteins form interconnected disc-shaped molecular assemblies. Based on our studies, such disc-shaped molecular assemblies of PSD are likely formed via phase separation. Importantly, the PSD assemblies are highly dynamic in response to synaptic activity changes. Mechanistically, the dynamic PSD assemblies are regulated by various enzymes such as Ca^2+^-calmodulin protein kinase II and protein phosphatases.

***Poo:*** Are there phase separation events at the presynaptic terminal?

***Zhang:*** Yes. In mature synapses, the presynaptic structures are fairly stable and contain a huge number of synaptic vesicles (SVs). These SVs mostly exist in two pools—a reserve pool in the presynaptic bouton, and a readily releasable pool docked on the active zone right above the presynaptic membrane. Both pools are condensed phase organized by phase separation, and SVs can exchange between these two pools.

The reserve pool is mainly organized by a protein called synapsin, which can undergo phase separation to form a condensed phase. Moreover, synapsin directly interacts with the SV

For the field of biological phase separation, we urgently need proper theory to guide future studies.

—Mingjie Zhang

surface, thus clustering the SVs into the condensed phase, forming coacervated condensates.

On the other hand, the active zone is a condensate formed by a different set of proteins, including RIM and RIM-BP. SVs can physically coat the surface of RIM–RIM-BP, thus docking on the active zone. Actually, this phenomenon perfectly explains the observation that the number of docked SVs in each bouton is proportional to the area of active zone.

I think the presynaptic terminal is a wonderful example showing different modes of interactions and communications between membranous organelles and non-membranous organelles. In this case, they can also be regulated by physiological activity, namely the action potentials.

### Directions

***Poo:*** Phase separation can localize a large number of biomolecules in a very specific region of the cell. I guess many signal transduction pathways could be carried out in the separated phases. Is there any evidence for this?

***Zhang:*** There are actually a number of studies being done in this direction. For example, in receptor tyrosine kinase signaling, people have produced evidence that phase separation can help with pathway efficiency and specificity.

In our study, we found that two enzymes, GIT and β-PIX, interact with each other very strongly and specifically. In living cells, they are able to form condensates, via phase separation, that act as modular functional entities. In different cell types, the condensates respond to different upstream signals and perform different activities. In synapses, they bind to Shank; in cell-cell junctions, they bind to Scribble; and during cell migration, they respond to the integrin pathway by binding to paxillin.

I think phase separation is a very good way for cells to increase the efficiency and specificity of signal transduction. It will be surprising if cells do not use this kind of strategy more often than we have realized.

***Poo:*** What do you think will be the future direction of biological phase separation research?

***Zhang:*** I have always believed that any biological process has to obey physics laws. All the theories governing molecular interaction that we use in biology are based on dilute solution systems, but now, phase separation brings biological systems into the realm of soft matter physics. However, currently available soft matter physics theories break down in the living cells due to the complexity.

My own training in physics is limited, so I think moving forward, one of the very important directions for myself is that I need to collaborate with people who understand soft matter physics. For the field of biological phase separation, we urgently need proper theory to guide future studies.

***Poo:*** That's great. Protein–protein interaction and its spatial-temporal regulation is the key problem of cell biology. Phase separation came at the right time to broaden our understanding of this interaction. I congratulate you that you entered this very important area at the right time.

***Zhang:*** Yes, it's very fortunate that we stumbled into this area during our study, and we are working very hard to discover more physiological connections of phase separation.

## A NEW SCHOOL OF LIFE SCIENCES IN SOUTHERN CHINA

***Poo:*** You became the first Dean of the School of Life Sciences at SUSTech several months ago. What is your vision for this new school?

***Zhang:*** In the past 25 years, I have had an incredible amount of fun doing science and training younger-generation scientists. But in recent years, I felt that I might be able to contribute a little bit to our educational and research system. So, SUSTech really gives me a wonderful opportunity, as well as a huge number of challenges. Shenzhen has developed into such a prosperous city that can be compared to Beijing and Shanghai, but its research and educational capacity is left far behind. To support the development of southern China, we need to build a strong school of life sciences in Shenzhen. That is why I decided to move from Hong Kong to SUSTech.

I looked into various life sciences research institutions worldwide and consulted many of my friends around the world. After that, I decided that the structure of this new school should not follow traditional settings. We would create a new structure that can better integrate different research areas for better development of modern life sciences. We retained the Department of Biology from which the School of Life Sciences is derived, but did not set up the traditional departments of biochemistry, cell biology and so on. Instead, we set up four other departments, which are the Department of Immunology and Microbiology, the Department of Systems Biology, the Department of Chemical Biology and Quantitative Biology, as well as the Department of Neuroscience. These are all important and interdisciplinary frontiers of life sciences. Also, the boundaries between these departments are very soft. There will be extensive collaborative opportunities among scientists in all these departments.

It's also very important that we establish a research environment that is supportive, nourishing and stable for the researchers to perform fundamental studies. This will be extremely challenging because, currently in China, young scientists are under extremely high pressure to produce publications within a short time duration.

I’m quite ambitious. I hope in 10 or 15 years, the School of Life Sciences in SUSTech will be the best life sciences research

We will not evaluate researchers only by their publications, but by trustworthy peer evaluation of the importance and potential impact of their work.

—Mingjie Zhang

organization in the whole of southern China, including Hong Kong. Actually, I promised myself that this will be my first and my last administrative job. I will not take another one in the future, so I am very committed to building a strong School of Life Sciences in SUSTech.

***Poo:*** That's indeed very ambitious. What do you think is the biggest challenge in achieving your goal?

***Zhang:*** It's a great challenge to convince the university and the governmental departments to be more patient, to provide sufficient support, and to give us more time and freedom to conduct the fundamental science without demanding quick results.

***Poo:*** I think it's also difficult to convince young scientists to address the important frontier problems, rather than following the hot fields.

***Zhang:*** Yes, I totally agree. This reality is largely due to our current evaluation system. I am trying to convince my colleagues that, in the long run, we will not evaluate researchers only by their publications, but by trustworthy peer evaluation of the importance and potential impact of their work. To do this, we need to establish an evaluation system that can be trusted by both the researchers and the administrators. This is something that is very difficult but that we have to achieve.

I hope that in 10 or 15 years, when people talk about some of the biological research areas, they will think of researchers in SUSTech because they have made notable contributions. That will have a real impact on science and on the world.

***Poo:*** Running a school of life sciences is extremely time-consuming. How are you going to balance your own research and administrative work?

***Zhang:*** That's very tough. If there is any downside to taking this job, it's that I have to considerably scale down my own research. But I think I have no choice but to take this responsibility. If I can help my colleagues to develop their careers, and help life sciences in SUSTech to be competitive, it's probably worthwhile.

## Notes

Weijie Zhao is an NSR news editor based in Beijing; Mu-ming Poo is Director of the CAS Center for Excellence in Brain Science and Intelligence Technology, and Executive Editor-in-Chief of NSR.

